# The importance of urban natural areas and urban ecosystem services during the COVID-19 pandemic

**DOI:** 10.1371/journal.pone.0243344

**Published:** 2020-12-17

**Authors:** Nelson Grima, Will Corcoran, Corinne Hill-James, Benjamin Langton, Haley Sommer, Brendan Fisher

**Affiliations:** 1 Environmental Program, Rubenstein School of Environment and Natural Resources, University of Vermont, Burlington, Vermont, United States of America; 2 Gund Institute for Environment, University of Vermont, Burlington, Vermont, United States of America; Sveriges landbruksuniversitet - Campus Umea, SWEDEN

## Abstract

Urban, peri-urban forests and other natural areas provide a wide range of material and non-material benefits to people known as ecosystem services. Access to these areas has been linked to benefits for physical and mental health of local populations. In the spring of 2020, the COVID-19 global pandemic forced many governments to impose a set of restrictions including the closure of businesses, cancelation of public events and schooling, social distancing, limitations on the size of social gatherings, and travel restrictions. During this period of restrictions, we conducted a study assessing the importance of urban and peri-urban forests and other natural areas to people living in and around the city of Burlington, Vermont, USA. We evaluated the self-reported use and changes in personal importance related to these natural areas before and during the period of restrictions. We received over 400 responses to our field survey. The results show that 69.0% of the respondents had *increased* or *greatly increased* their visitation rate to our natural areas and urban forests, and 80.6% of respondents considered that the importance of these areas, and access to them, either *increased* or *greatly increased*. Moreover 25.8% of the sample had either never, or very rarely accessed their local natural areas before the pandemic, but 69.2% of the first time or infrequent visitors reported that having access to these areas during COVID-19 as ‘very important’. People reported that these areas were important for a wide range of activities from exercise to birding, but also reported values related to reducing stress in a time of global chaos. Our results indicate the increasing demand and value of such areas in times of crisis such as COVID-19. Experts in zoonotic disease predict the potential for more frequent pandemic events, thus predicating the importance for continued funding for, maintenance of, and improved access to, natural areas to our largely urban civilization.

## 1. Introduction

The SARS-CoV-2, also known as COVID-19, is a viral infectious disease that affects mainly the human respiratory system [1] and was first identified in early December 2019 in Wuhan, Hubei province, China, and since then, it has spread rapidly becoming a global pandemic [2]. As of September 2020, the pandemic has caused over 950,000 deaths globally [3], and it is expected that, despite the rate of infections slowing down, deaths will continue to occur throughout this year [4]. Although it is not possible to provide an accurate estimation of global economic losses due to the pandemic, the International Monetary Fund has estimated that the global economy will contract at least 3% in 2020—a figure larger than the 2008–2009 global recession. Governments across the globe have issued mobility restrictions aiming to stop the spread of the disease, resulting in approximately half of the world’s population to stay at home and avoid public spaces [5]. Besides the economic impact of this situation, there are increasing concerns regarding the less obvious effects on mental health. These effects are derived from isolation, disconnection, unemployment, stress, etc., and may result in long term impacts beyond the time frame of the current pandemic [see for example 6].

We were interested in how people are using natural spaces to cope with the physical and mental health challenges, and feelings of isolation, predicated by ‘stay at home’ orders and social isolation. A growing literature has produced an evidence base suggesting that nature exposure helps to deliver mental health benefits [7], reduced stress levels [8], less rumination (i.e. the dwelling on negative aspects of a recent situation) [9], and increased overall sense of wellbeing [10]. Not only does outdoor recreation and exercise have a positive impact on mental health, but physical health as well. A systematic literature review found that outdoor exercise, or ‘green exercise’ leads to increased fitness, better cardiovascular function, reduced blood pressure, and has a positive influence on several other health markers [11]. A study conducted on elderly citizens in Tokyo even found that regular walking in green spaces, compared to more industrial settings, increased life expectancy [12]. Compared against indoor sports and exercise, outdoor activities in which people are surrounded by nature, have proved to have a greater positive impact on physical health [13].

Beyond the physical and mental health benefits, the outdoors and green spaces contribute to making people act more positively in social settings, setting a context to improve social relations and overall wellbeing [14–17]. For example, outdoor social settings in which people are surrounded by greenery while having face to face conversations, have proven to increase social cohesion [14]. Evidence shows that greenery in urban settings is associated with less violence and aggressive behavior [15–17], and more generally, some studies suggest that the presence of healthy vegetation and access to green spaces improve people’s well-being and welfare [e.g. 18, 19].

There is a smaller literature on the importance of access to nature in times of economic and social crises. For example, during the 2008 recession, American media saw a large uptick in the amount of coverage for urban foraging [20]. As millions of Americans lost their jobs and were pushed into food insecurity, some relied on their urban greenspaces to provide sustenance and a mental escape from the stressful times [20]. Following the 2011 earthquake, tsunami and nuclear disaster in Futaba County, Japan, city officials have made it a point to rebuild key green spaces that provide cultural ecosystems services crucial to the recovery of locals [21]. These green spaces are crucial to providing a sense of pride and identity for residents as they recover from this disaster [21]. A systematic literature review also uncovered how interactions with ecosystems and the cultural services they provide are greatly beneficial for refugee populations as they recover from the physical and emotional upheaval during resettlement [22].

Focusing on urban (within a metropolitan area) and peri-urban (immediately adjacent to a metropolitan area) natural areas, studies show the wide range of benefits they provide to people [23]. These benefits are known as ecosystem services, and they can be divided at large between material and non-material benefits [24]. Some of the non-material benefits derived from the interactions between humans and the ecosystems (e.g. spiritual fulfillment, recreation, aesthetic experiences, cultural heritage) are defined as Cultural Ecosystem Services [25].

Cultural Ecosystem Services (CES) are particularly important in urban natural areas, where the aims of management tend to be towards fulfilling non-material needs rather than providing materials. There are different approaches to enjoy these services. For example, services such as enjoyment of aesthetic beauty, sense of place, or recreation, can be enjoyed actively (e.g. doing sports) and/or passively (e.g. observing nature from a fixed point) from being able to access urban parks and forests or other natural areas near towns [26]. The importance of enjoying these benefits is such that it has been linked to physical and mental health benefits, in particular for communities living in large urban concentrations [27–29]. In this study, we focus on how people living in the largest metropolitan area of the state of Vermont, USA, make use of the urban and peri-urban natural areas of the region and benefit from CES during the COVID-19 social restrictions. Our hypothesis was that these spaces were more utilized and important to locals during this global pandemic. Our goal was to add to the evidence base that tests the importance of access to nature, and the importance of CES during times of crises [20–22].

In this state, COVID-19 was first identified on the 7^th^ of March, 2020 [30]. The subsequent spread of the disease throughout the state triggered the government to declare a State of Emergency on the 10^th^ of March 2020, and to issue a set of restrictive measures aimed to slow down and help to contain the spread of the disease [31]. These measures included the closing of non-essential businesses, schools and universities, the prohibition of mass gatherings, and the official order to all Vermont residents to stay at home leaving only for critical reasons related to health and safety. Nevertheless, undertaking outdoor activities such as walking in natural areas remained allowed as long as social distancing (approximately 1.5 m distance between people not sharing a household) was maintained [32].

During this time, we noticed an increase of visitors to urban parks and forests in Burlington, Vermont. The perceived increase in use of urban parks and forest areas was likely driven by the shutdown of all other social and market options. However, we wanted to evaluate this perceived increase in use, and better understand the values expressed by the users of these areas in this time of pandemic, understanding ‘value’ as it is considered subjectively by the participants in this study. As such, we launched a survey focused on 25 urban and peri-urban natural areas in and around the city of Burlington, Vermont. Our aim was to better understand how urban and peri-urban natural areas help to provide the non-material basic needs of urban communities (e.g. a place to enjoy nature, exercise or recreate in the open air), and we explore how the benefits and values these areas provide to people change during times of crisis like we now find ourselves in.

## 2. Methods

### 2.1. Study sites

The University of Vermont (UVM) owns and manages ten natural areas across the state of Vermont [33, 34]. These areas are used for academic purposes, but are also open to the general public for recreation. Seven of these natural areas are in the immediate vicinity of the city of Burlington and its metropolitan area, which concentrates roughly 214,000 people, approximately one third of the total population of the state [35]. There are other parks and natural areas in and around the city of Burlington managed by different entities such as Burlington Parks, Recreation & Waterfront (BPRW), the Intervale (I), the Episcopal Church in Vermont (ECV), or the Winooski Valley Park District (WVPD).

For this study, we focused on the seven natural areas managed by UVM that lay within the immediate surroundings of the Burlington metropolitan area and 18 parks and natural areas managed by other entities in the same area. These 25 parks and natural areas are the urban and peri-urban natural areas of Burlington. All 25 areas have hiking/walking trail systems within them, some more extensive than others. Each one of these areas is characterized by a different set of ecosystems, geomorphology, and species compositions. However, in general terms, the main ecosystems found across these areas are peatlands, swamps, wetlands, and open waters; forests of conifers, broadleaved trees, and mixed forests; and open fields, bushlands, and some agricultural lands.

### 2.2. Survey design and implementation

We designed and implemented an online survey to collect information from users of 25 urban and peri-urban natural areas located in and near Burlington, Vermont (see section 2.1). The survey was conducted in two phases between March 28^th^ and June 8^th^, and was released within three weeks of the initial spread of the COVID-19 pandemic and the concordant stay-at-home official orders in Vermont. The survey was completely anonymous, and consisted of 10 questions combining multiple-choice and open-ended questions, regarding the use of, and value of, the urban and peri-urban natural areas to local users and how these use and values interact with restrictions imposed by the COVID-19 pandemic. In order to increase anonymity and make the survey even quicker to fill in, we did not ask demographic questions such as gender or age. The online survey could be completed in less than five minutes, and access to it was made public through information sheets at the beginning of the main walking trails and entrance ways to these areas. The information sheets contained a brief explanation of the purpose of the survey, a QR code and a website address to access the survey. Upon accessing the online survey, the participants encountered a detailed explanation of the aims of the survey, as well as complete information on how the data would be managed. The ethical standards of this study were approved by the University of Vermont’s Research Protections Office and the Committees on Human Subjects (UVM STUDY00000892). After one month, a link to the website address containing the same survey and information was then sent out to various list servers linked to these natural areas (e.g. Burlington Parks and Recreation—who manage many of these areas—sent the survey to their subscribed patrons). These lists are composed of email addresses of people who subscribe to the servers in order to receive news, updates, etc. regarding the natural areas within Vermont. Roughly half of the sample accessed the survey from the trailheads. The other half of the sample accessed the survey through a weblink sent out to various list serves that serve the different park managers. This two stage approach may have helped to include frequent prior to COVID-19 visitors of the natural areas that either reduced or stopped their visits during the pandemic’s initial phase.

### 2.3. Data composition and analysis

The type of data we collected aims to be straightforward to provide the maximum amount of useful information with the minimum questions. The questions asked related to:

- Natural areas regularly visited (to discern possible preferences)
- Frequency of visits prior and during COVID-19 (to compare ‘before’ and ‘during’ frequencies)
- Personal importance of the natural areas visited and its change during COVID-19 (to better understand people’s attachment to the natural areas)
- Group size prior to and during COVID-19 (to observe if social-dynamics in nature have changed, since, ‘socializing’ in nature has been linked to livelihood improvements, and at the same time limiting ‘socializing’ has been one of the key measures during this pandemic)
- Key reasons for visiting (to understand what people consider important from these areas)
- Main residence zip code (to evaluate how far people travelled to visit the areas)
- Question open to any comments (to collect any information considered relevant by the respondents)

This study is based on a descriptive analysis of the data. Initially, we collected separately the responses from the trailheads and the responses from the email lists. We cleaned and harmonized the data across both sets of surveys and checked for redundancies. We analyzed how often respondents visited these natural areas prior to the COVID-19 pandemic and how their self-reported rate of visitation changed after the imposed restrictions. We compared how important these areas were to respondents both before and during COVID-19 restrictions. We evaluated the key reasons why the respondents visit these areas and the differences in the patterns of group size visiting these natural areas prior to COVID-19 and since the pandemic started.

## 3. Results

In the ten weeks of survey data collection (March 28—June 8) we received 346 valid responses representing visits to our 25 urban and peri-urban natural areas and parks. Comparing the two groups of respondents (data collected from the trailheads vs. data collected from the email lists), we observed that responses were very similar regarding the change in frequency of visits, where most of the respondents of both groups declared having increased or greatly increased their visits; the personal importance of these natural areas, where almost all respondents in both groups chose the option ‘important’ or ‘very important’; the change of importance of these areas after the restrictions took place, having mostly ‘increased’ or ‘greatly increased’ for both groups; and the reasons given for visiting the areas, being the most repeated reasons in both groups ‘just getting outside’, ‘exercise’, ‘connecting to nature’, and ‘peace and quiet’. The biggest differences observed between the two groups correspond to the frequency of visits prior to COVID-19, where the email lists group answered mostly that they visited 1–2 times per week, and almost nobody answered that they had never visited the areas, and the trailheads group had a more even distribution of responses across the choices given, and a considerable number of people declared that they had never been in the areas before. The other considerable difference among the two groups of responses relates to the group sizes prior to COVID-19, where the group from the email lists mainly visited alone or with one other person, and the group from the trailhead had a larger proportion of bigger groups.

**Fig 1:**
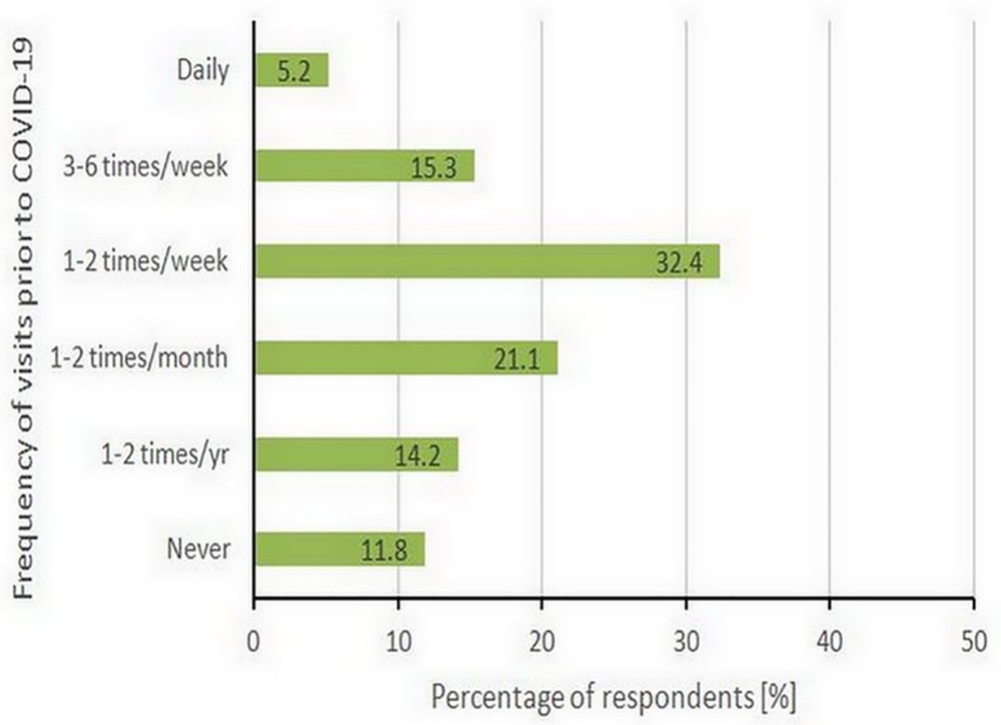
Frequency of visits to the local natural areas prior to the COVID-19.

Considering all responses together, prior to the COVID-19 pandemic about half of the respondents (52.9% of the sample) visited these natural areas either daily or weekly, while the other half either visited the areas monthly (21.1%), yearly (14.2%), or had never been there (11.8%) (Fig 1). During the survey period, over a quarter of the respondents (26%) never visited or were seldom visiting (i.e. once per year) the areas prior to COVID-19.

**Fig 2:**
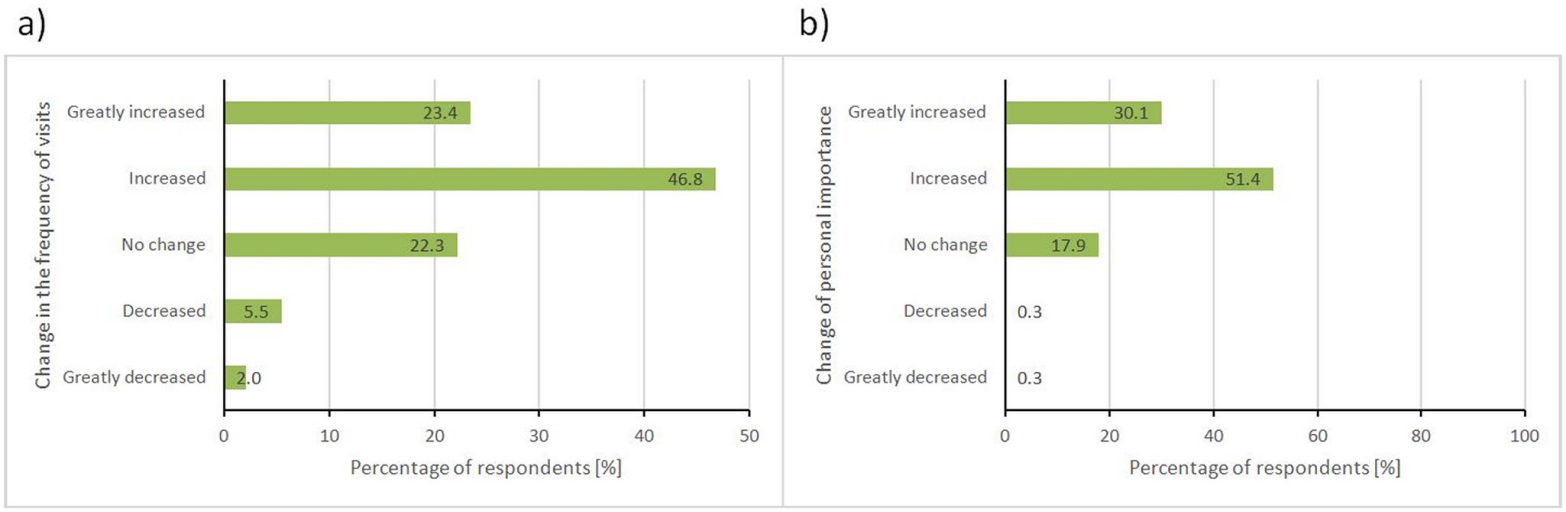
Changes in frequency of visits to natural areas (a) and personal importance of these areas (b) during COVID-19 compared to prior to COVID-19 times.

Almost half of the sample (46.8%) said they had “increased” their visitation rates to these natural areas, and an additional 23.4% claimed to have “greatly increased” their visitation rate, making a combined total of 70.2% (Fig 2a). A minority of the sample (5.5%) reported their use of the natural areas had decreased or greatly decreased (2.0%), and the remaining 22.3% reported no change in their use of the natural areas during the first 10 weeks of the pandemic. The respondents who most increased their rate of visits to these natural areas were those who prior to COVID-19 where already visiting these areas once or twice per week, and once or twice per month, followed by those who had never visited the areas before. The biggest decrease in visits corresponds to the frequent visitors, who were visiting these areas three to six times per week prior to COVID-19. In addition to a large reported increase in use of natural areas in this time of pandemic, respondents also reported that their personal importance of being able to access these areas also changed. The majority of respondents said that the importance of these natural areas to them either “increased” (51.4%) or “greatly increased” (30.1%) during pandemic times, resulting in a combined total of 81.5% (Fig 2b). Only two respondents reported that the value of these areas had decreased for them, with one of the respondents indicating in an open-ended comment that they “don’t trust people to be respectful of physical distancing” in the natural areas.

**Fig 3:**
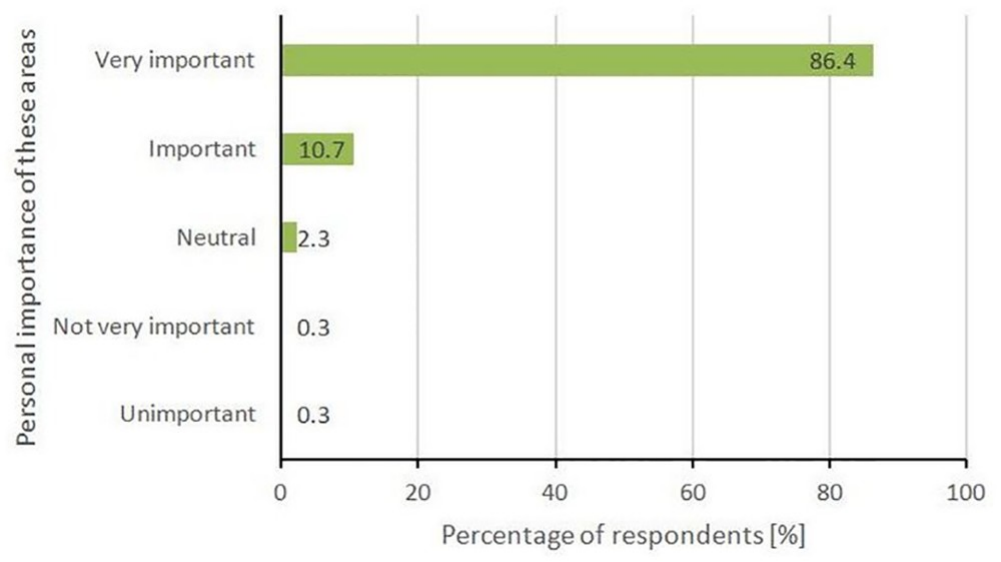
Importance of visiting natural areas during COVID-19.

The majority of this convenience sample considered that these natural areas are either ‘very important’ (86.4%) or ‘important’ (10.7%), with only two people (0.6%) considering these areas not important to them (Fig 3).

**Fig 4:**
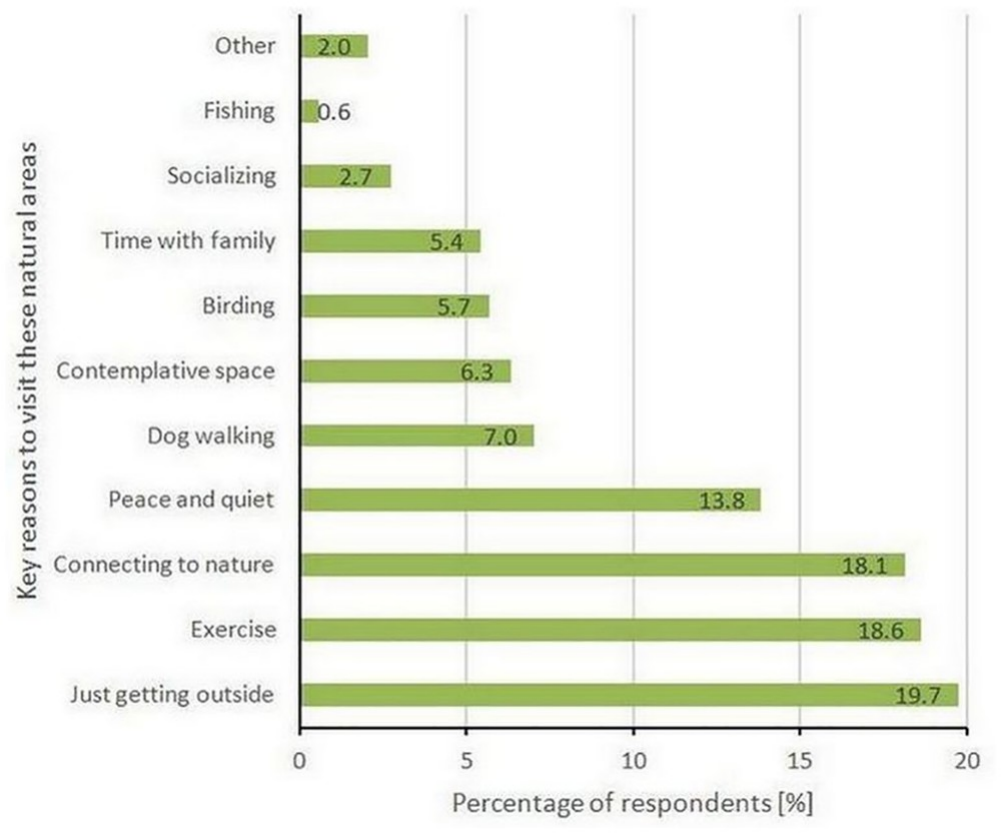
Key reasons, as stated by the respondents, for visiting the natural areas under study.

We asked the respondents what motivates them to visit these areas. The respondents had the opportunity to check off as many pre-defined reasons as appropriate, but also to add their own reasons. There were over 30 unique responses to this question, but the most common answers included simply getting outside, exercise, connecting to nature, finding peace and quiet, finding contemplative space, dog walking, time with children and birding (Fig 4).

**Fig 5:**
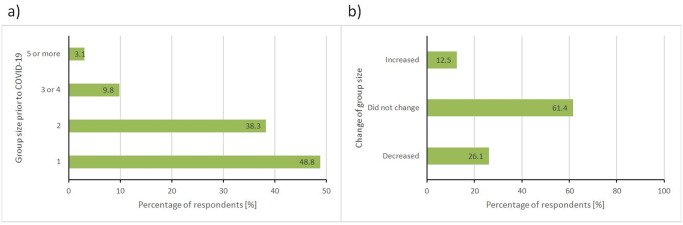
a) Differences in the group sizes visiting these natural areas prior to COVID-19, and b) Changes in the pattern of group size visiting these natural areas since the COVID-19 pandemic began.

Finally, we wanted to see if the pandemic had changed the pattern of how people visit these areas with respect to the size of the groups they visit with (Fig 5). Prior to COVID-19, respondents predominantly visited these places alone (48.8%) or with one other person (38.3%). For the majority (61.4%), the pandemic did not change the pattern of their visitorship. However, for 26.1% of the sample, COVID-19 drove respondents to decrease their party size in these areas. Interestingly, for 12.5% of the sample, the size of their groups increased during COVID-19. Remarkably, most of the people (80.0%) who used to go alone to the natural areas prior to COVID-19, continued to do so, while all people who declared going previously in groups of five or larger are now going in smaller groups. People who were already going in groups of two or three are the ones with more variability in the present group size. However, there is no clearly defined pattern among them.

## 4. Discussion and conclusion

In this study, we designed and implemented a survey to collect information regarding the use of CES from urban and peri-urban natural areas, and how the personal importance that people attribute to them change in times of social crisis (e.g. under the restrictions imposed due to the COVID-19 pandemic). Our rapid, descriptive case study provides empirical evidence in support of other evidence (e.g. data collected by private companies via cell phone localization), that the need for, and use of, nature has increased during this time of pandemic [see 36].

We found that people in our survey increased the frequency of their visits to natural areas during the COVID-19 social distancing restrictions, a result that aligns with other studies’ observations in other parts of the world [e.g. 37, 38]. This increase included about 26% of respondents who would be considered rare-to-first-time visitors. While the majority of the sample continued to visit these spaces with the same size groups as prior to COVID-19 (typically alone or with another one person), slightly over a quarter of the sample reduced the size of their visiting group during COVID-19 and 12.5% increased the size of their group. These results can imply that 1) for the majority of the sample social-distancing or group size restrictions did not affect their typical habits 2) for some of the sample they decreased their sociality during visits (i.e. smaller numbers) and 3) some of the sample used the natural areas as a safe space for increasing their social interactions via use natural areas. Evidence for this last assertion are open-ended comments referring to how easy it is to spend time with others while maintaining the social distance. All of these dimensions will be important as the pandemic continues or more come down the line—i.e. people will want to continue some parts of their lives as business as usual; people will need more space (e.g. for peace, contemplation etc.); people will need safe spaces to be social when so many other outlets are closed to them.

Our respondents also indicated that COVID-19 has forced them to re-evaluate and elevate their importance for such spaces. 81.5% of respondents reported that the importance of such spaces to them *increased* or *greatly increased* since the onset of COVID-19 social and work restrictions. This increase in importance supports recent observed findings from twitter analysis that people visiting urban green spaces, such as the ones investigated here, have lower negativity and higher sentiment and that visits to such places could have effects that extend beyond parks limits [39]. In our survey 38.2% of people reported that they utilized these natural areas to find peace and quiet, to connect to nature, or to use these areas as contemplative spaces—activities that have been shown to reduce stress and rumination. As such, access to our urban and peri-urban natural areas may be delivering these mental health precursors during a time when they are most needed. This is reflected on the recommendations that other researchers have posed to their governments [e.g. 40].

If our results hold true in other regions, demand for urban and peri-urban greenspace is increasing just at the time we are seeing losses of urban and peri-urban natural areas or vacillating priority for such areas in parts of China [41], and the United States [42]. In addition to the potential loss of such spaces in times of need, we know that in many places, access to urban green space is unequal with several US cities showing that access to urban green space is a function of income and race, i.e. negatively correlated with poor and being a person of color [43]. Given that COVID-19, at least in the USA, is more likely to negatively impact people in lower income brackets, the lack of access to green space may compound the more diffuse, yet pernicious effects of the COVID-19 pandemic [26].

In our study, respondents indicated over 30 unique activities and drivers of their welfare provided by natural areas, from the most common “just getting outside” to the deeper and more abstract statements of those who find it important as a “contemplative space” in times of global chaos. The results also highlighted that many of the visitors to natural areas had either gone for the first time, or had been for the first time in months or years. Of the 41 respondents who had never been to any of these natural areas before COVID-19, 21 of them reported that they are now visiting these areas frequently. Our study was not designed to evaluate the persistence of importance to users over time, however, 29% of the sample were either interested in the results, or provided positive support for the existence and access to the natural areas by offering their emails and offering open-ended comments. We suggest this indicates a deeper engagement with these areas than simply responding to a survey.

Our study was conducted in Vermont, a state known for its relatively low population density, extensive forests and natural landscapes, and a high number of people that link their leisure time to outdoor activities [44]. Thus, a similar study implemented in regions with higher population densities or with other attitudes toward nature, might yield somewhat different results. Our sample is a convenience sample of people visiting the natural areas and is not representative of the wider population, and carries with it the limitations of self-reported data [45]. Moreover, the distribution of responses is not uniform across the 25 areas under study (see S1 Data). As such our approach has likely biased the sample towards over-representing frequent visitors of these natural areas, and certainly biased this sample by missing many of those visitors that have stopped visiting during COVID-19. Future research using a larger sample and a longitudinal study approach could test the strength of our results, and whether the findings are valid throughout the duration of the pandemic. This study did not address issues related to income, education, or race which are certainly important avenues of inquiry for further studies.

Given the time constraints we faced to design and implement the survey, we committed errors in the design of a couple of questions. One of these errors is in the question asking about the frequency of visits, where we gave different options but there is a significant gap between the options of 1–2 times per month and 1–2 times per year. We realized that some visitors might have benefited from an intermediate option between the two mentioned. The other one of these errors is in the measurement scale used for the question where we asked about the importance of the natural areas. The scale is not exactly even on both sides, and while we included “important” and “very important” on the positive side, we included “unimportant” and “not very important” in the negative side instead of “unimportant” and “very unimportant”. Although respondents could intuitively provide an adequate answer, technically the scale should have been correct.

Global pandemics such as COVID-19 are predicted to increase in frequency in the future [46]. Our results show that people seek out urban and peri-urban natural areas to help mitigate the adverse impacts through the provision of places and spaces that can deliver non-material ecosystem services and human welfare benefits in a time of crises. Purported health benefits of access to, and use of, urban and peri-urban natural areas have included cardiovascular health [47], mental health outcomes [48], improved pulmonary function [49], in so-called normal times. Other authors have studied the effects of using nature as a therapy for physical and mental disorders [e.g. 50, 51], or have analyzed the role of natural settings for individual crisis rehabilitation [e.g. 52]. We will need longer term research and more direct observational and biometric-based studies to get a deeper handle on the differential impacts of such spaces during times like COVID-19, however it seems safe to assume that demand for, and the beneficial effects of, such spaces on physical and mental health can only increase. Future research could focus on i) the differential effects of benefits across people of different agency, income, race and culture ii) the justice and equity in access to such benefits iii) the interactions between the benefit of urban and peri-urban natural areas and the various impacts of future pandemics (isolation; cardiovascular health; unemployment; depression) and iv) the persistence of these health and non-material benefits to new ‘users’ of natural areas. Efforts in this direction have already begun [e.g. 53].

The increased use of urban and peri-urban natural areas may lead to conflicts or tradeoffs in use, space and values. Thus, a planning and management of these areas that strives for equal accessibility or foresees and avoids possible crowding of specific sites is essential. The United Nations World Tourism Organization released a series of general guidelines aimed to establish a common understanding of how to manage natural areas ensuring that visitors do not undermine their values [54]. However, each natural area has its own characteristics, and needs its own tailored management. A basic need for such planning is to monitor the spatial and temporal flows of visitors to these areas [55]. Knowledge on visitor flows provides grounds to establish a planning of these areas that takes into account the a) environment, b) economics, c) enforcement of regulatory measures, d) experience and visitors satisfaction, e) engagement of the local communities, f) technical and social implications of visitors use, and g) education regarding the natural and cultural environments [56].

Urban and peri-urban natural areas have been shown to be important in ‘normal’ times [57, 58] and in times of crisis [20, 21] and upheaval [22]. The popular press has touted the importance of outdoor space and nature in these globally stressful times [e.g. 59]. Here we provide some empirical evidence to demonstrate just how valuable urban and peri-urban natural areas are for local welfare in such times. Our results corroborate observations on the importance of natural areas in time of crises. With the specter of future crises like COVID-19, natural areas and ecosystem management budgets should be safeguarded and potentially increased as a tool to maintain and improve human welfare in times of crises, even as we see major contractions in our economic foundations.

## Supporting information

S1 Data Data collected during the survey. (XLSX)

S1 File Survey questions. (PDF)
