# Supplementary material for: The importance of urban natural areas and urban ecosystem services during the COVID-19 pandemic
Source: PLoS One. 2020 Dec 17;15(12):e0243344. doi: 10.1371/journal.pone.0243344 (PMC7746267; doi:10.1371/journal.pone.0243344)
Supplement: S1 File — (PDF) [file pone.0243344.s002.pdf]

Natural Areas Survey Questions

1. Which natural areas do you regularly visit?

2. Before COVID-19, how often would you typically visit these areas?

□ Never □ 1-2 times per year □ 1-2 times per month □ 1-2 times per week

□ 3-6 times per week □ Daily

3. Has the frequency of your visits changed since the COVID-19 pandemic?

□ Greatly decreased □ Decreased □ No change □ Increased □ Greatly increased

4. How important are these natural areas to you?

□ Unimportant □ Not very important □ Neutral □ Important □ Very important

5. Has the importance of visiting natural areas changed for you since the COVID-19 pandemic?

□ Greatly decreased □ Decreased □ No change □ Increased □ Greatly increased

6. Before COVID-19, how many people would you typically visit these areas with, INCLUDING yourself?

□ 1 □ 2 □ 3-4 □ 5 or more

7. Has the number of people you visit with changed since the pandemic?

□ Decreased □ Did not change □ Increased

8. What are the key reasons or values for your visits to these natural areas? (Check as many as apply)

□ Just getting outside / Fresh air

□ Exercise

□ Connecting to nature

□ Peace and quiet

□ Dog walking

□ Contemplative space

□ Birding

□ Time with family / children

□ Socializing

□ Fishing

□ Other _________________________

9. It would help in our work if you could provide the zip code of where you currently live:

10. Do you want to say anything else? If you would like to see the results of this survey, you may provide your email address.
